# Clinical application of transcranial neuroendoscopy combined with supraorbital keyhole approach in minimally invasive surgery of the anterior skull base

**DOI:** 10.1038/s41598-024-65758-y

**Published:** 2024-06-27

**Authors:** Long Zhou, Xiongfei Jing, Chang Wang, Huikai Zhang, Pan Lei, Ping Song, Zhiyang Li, Lun Gao, Minghui Lu, Qianxue Chen, Qiang Cai

**Affiliations:** 1 Department of Neurosurgery, Renmin Hospital of Wuhan University, No. 238, Jiefang Road, Wuchang District, Wuhan City, 430060 Hubei Province China; 2 Department of Neurosurgery, Xiantao First People’s Hospital Affiliated to Yangtze University, No. 29, Middle Section of Mianzhou Avenue, Xiantao City, 433000 Hubei Province China; 3 Department of Neurosurgery, Xiaochang First People’s Hospital, No. 1, Zhanqian Road, Xiaochang County, Xiaogan City, 432900 Hubei Province China

**Keywords:** Transcranial neuroendoscopy, Supraorbital keyhole approach, 3D slicer, Cerebral aneurysm, Brain tumor, Frontal lobe cerebral hemorrhage, Cerebrospinal fluid rhinorrhea, Diseases of the nervous system, Anatomy, Neurology, Oncology

## Abstract

To explore the techniques, safety, and feasibility of minimally invasive neurosurgery through the supraorbital eyebrow arch keyhole approach by neuroendoscopy. Retrospective analysis of clinical data of patients with various cranial diseases treated by transcranial neuroendoscopic supraorbital eyebrow keyhole approach in our hospital from March 2021 to October 2023. A total of 39 complete cases were collected, including 21 cases of intracranial aneurysms, 9 cases of intracranial space occupying lesions, 5 cases of brain trauma, 3 cases of cerebrospinal fluid rhinorrhea, and 1 case of cerebral hemorrhage. All patients’ surgeries were successful. The good prognosis rate of intracranial aneurysms was 17/21 (81%), and the symptom improvement rate of intracranial space occupying lesions was 8/9 (88.9%). Among them, the initial symptoms of one patient with no improvement were not related to space occupying, while the total effective rate of the other three types of patients was 9/9 (100%). The average length of the craniotomy bone window of the supraorbital eyebrow arch keyhole is 3.77 ± 0.31 cm, and the average width is 2.53 ± 0.23 cm. The average postoperative hospital stay was 14.77 ± 6.59 days. The average clearance rate of hematoma by neuroendoscopy is 95.00% ± 1.51%. Our results indicate that endoscopic surgery through the supraorbital eyebrow arch keyhole approach is safe and effective for the treatment of anterior skull base lesions and cerebral hemorrhage. However, this retrospective study is a single center, small sample study, and the good surgical results do not exclude the subjective screening of suitable patients by clinical surgeons, which may have some bias. Although the clinical characteristics such as indications and contraindications of this surgical method still require further prospective and multicenter clinical research validation, our study still provides a new approach and choice for minimally invasive surgical treatment of anterior skull base lesions.

## Introduction

With the advancement of medical imaging technology, neuronavigation technology, surgical optical equipment, and surgical techniques, the pursuit of neurosurgeons to reduce surgical treatment related side injuries seems to have become a trend. This has led neurosurgeons to design a “minimally invasive” surgical approach that minimizes surgical trauma while also considering cosmetic effects. The concept of “minimally invasive” surgery is to minimize ineffective exposure of the surgical area and minimize harassment of non-target brain tissue during the operation process. Based on the above concepts, neurosurgeons have developed a supraorbital eyebrow arch keyhole approach to enter the surgical area of the anterior and middle cranial fossa. With the assistance of neuroendoscopy, surgeons can obtain the maximum surgical area through the smallest surgical channel, while optimizing the surgical effect to maximize patient benefits^1^. With the development of surgical optical equipment and the demand for high-quality lighting in the surgical area by surgeons, neuroendoscopic technology is increasingly favored by neurosurgeons. Neuroendoscopy significantly enhances the high-quality lighting of the surgical area, providing a wider surgical field of view and higher magnification, as well as its ability to “look around”, clearly and concisely displaying the local structural details of the surgical area and its relationship with surrounding tissues^2^. The combination of supraorbital keyhole approach and neuroendoscopy perfectly embodies the concept of minimally invasive neurosurgery, fully leveraging the advantages of minimal surgical trauma, clear surgical field of view, good surgical results, short hospitalization time, low total hospitalization costs, and good cosmetic effects^3–5^.

## Materials and methods

### General information

This retrospective study collected clinical data of patients with various cranial diseases who underwent surgery through the eyebrow arch keyhole approach under transcranial neuroendoscopy in our hospital from March 2021 to October 2023. A total of 39 complete cases (aged 29–76 years) were collected, including 21 cases of intracranial aneurysms, 9 cases of intracranial space occupying lesions, 5 cases of brain trauma, 3 cases of cerebrospinal fluid rhinorrhea, and 1 case of cerebral hemorrhage. All patients’ surgeries were successful.

### Typical cases

#### Case 1

**Figure 1:**
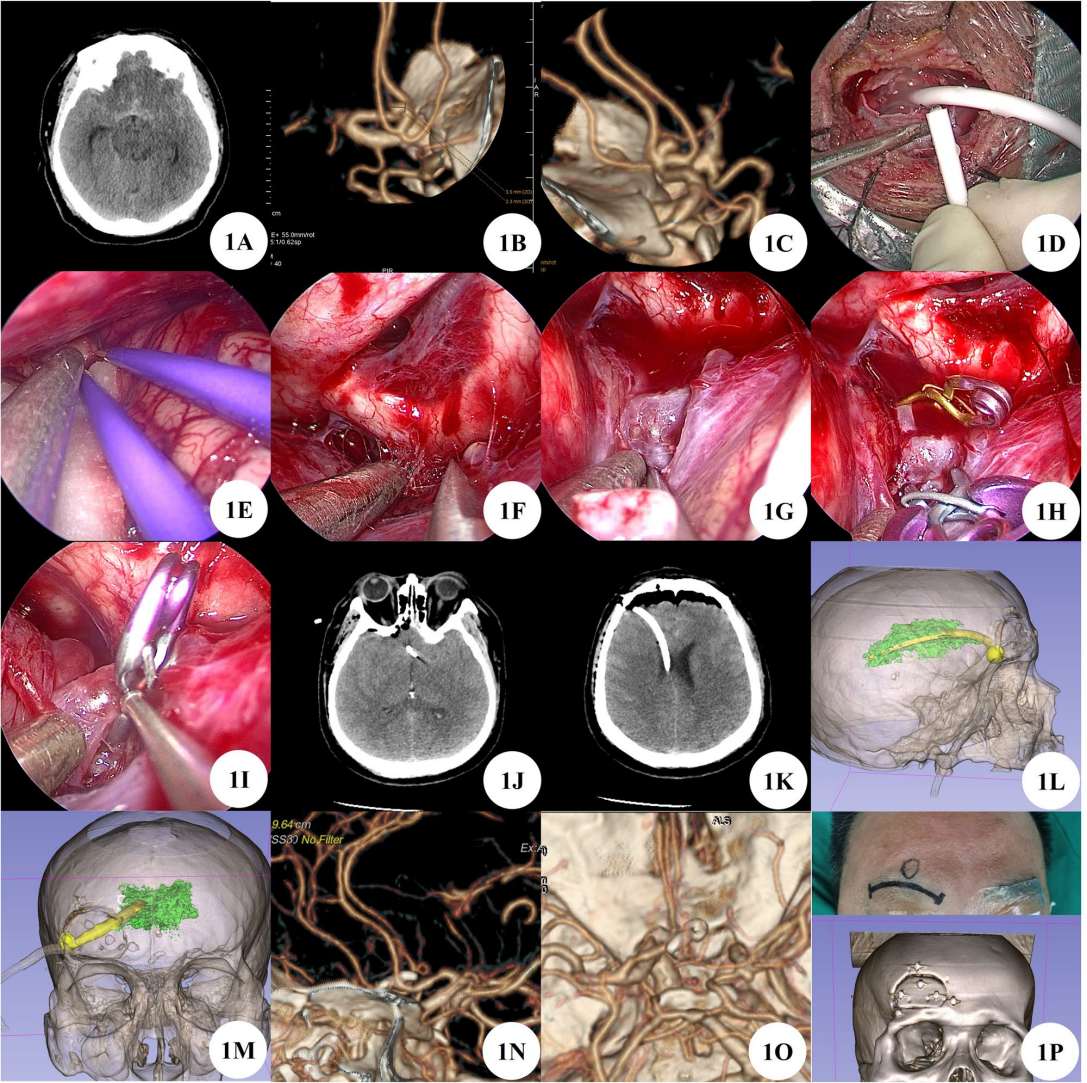
(**A**) Preoperative CT shows subarachnoid hemorrhage. (**B**–**C**) Preoperative CTA suggests anterior communicating artery aneurysm. (**D**) Intraoperative puncture of the lateral ventricle to release cerebrospinal fluid for decompression. (**E**–**I**) Separation and clipping of aneurysms by neuroendoscopy. (**J**) Postoperative CT shows aneurysm clip. (**K**) Postoperative CT scan revealed ventricular drainage tube. (**L**,**M**) Postoperative 3D Slicer reconstruction of virtual reality images of lateral ventricles and extraventricular drainage tubes. (**N**,**O**) Postoperative CTA indicates complete occlusion of the anterior communicating artery aneurysm, with no stenosis in the parent artery and branches. (**P**) Surgical incision and bone window.

A 49 years old male patient admitted the hospital for sudden severe headache. After admission, complete cranial CT showed subarachnoid hemorrhage, intraventricular hemorrhage, and diffuse brain swelling. Head and neck CTA shows an aneurysm of the anterior communicating artery and an aneurysm at the beginning of the P1 segment of the right posterior cerebral artery. Considering the size of the patient's intracranial aneurysm and the extent of subarachnoid hemorrhage, it is considered that the anterior communicating artery is a ruptured aneurysm with a high risk of rebleeding, and emergency surgical treatment is needed. After fully evaluating the surgical plan and risks, the right supraorbital eyebrow arch keyhole approach was chosen for surgical treatment. During the operation, severe swelling of brain tissue was observed, and puncture was performed to release cerebrospinal fluid for decompression in the lateral ventricle of the surgical field. Then, routine surgical treatment was performed, and the surgery was successful with good results. And interventional embolization was performed again at the beginning of the P1 segment aneurysm of the right posterior cerebral artery 24 days after surgery. One week after surgery, a follow-up head and neck CTA showed no significant residue of the anterior communicating artery aneurysm and the P1 segment aneurysm of the right posterior cerebral artery, and no delayed bleeding or stenosis of the parent artery. After one month of outpatient follow-up, the patient had clear consciousness and no positive signs of the nervous system (Fig. 1A–P).

#### Case 2

**Figure 2:**
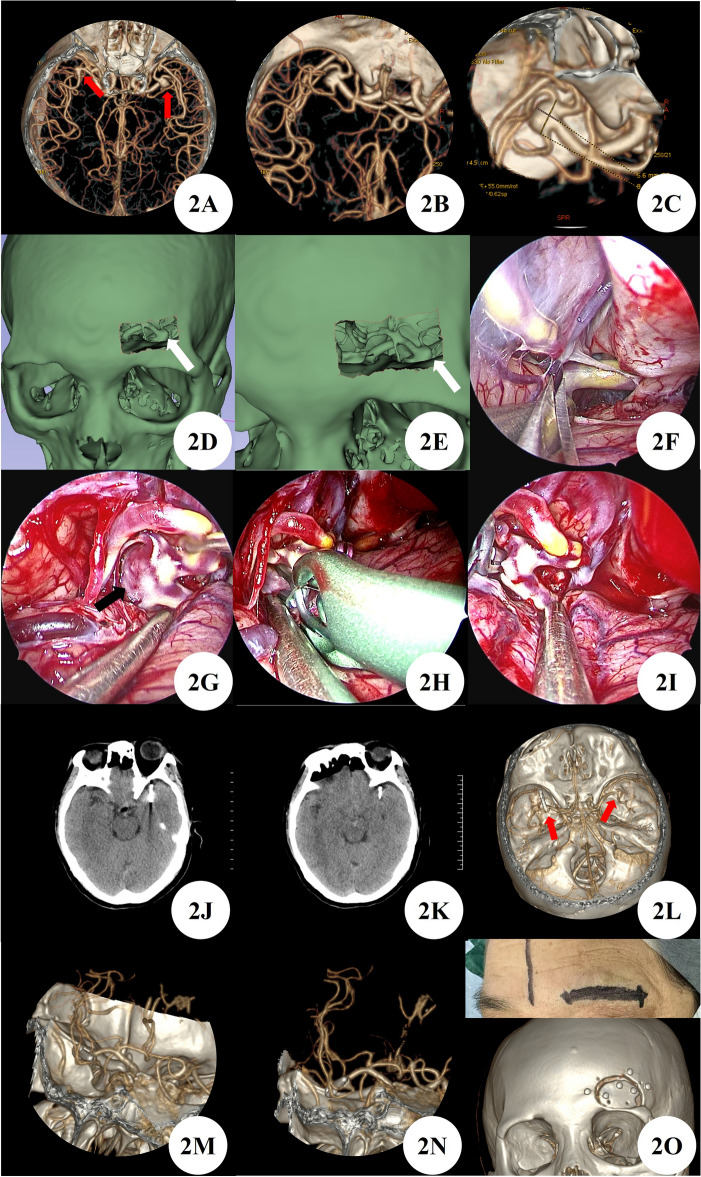
(**A**–**C**) Preoperative CTA showed bilateral middle cerebral artery bifurcation aneurysms and left middle cerebral bifurcation aneurysms that require priority treatment (red arrow). (**D**,**E**) Feasibility evaluation of supraorbital eyebrow arch keyhole surgery using 3D Slicer reconstruction virtual reality technology (white arrow). (**F**–**I**) Neuroendoscopic clipping of left middle cerebral artery bifurcation aneurysm. (**J**,**K**) Postoperative CT showed aneurysm clipped. (**L**–**N**) After clipping the aneurysm at the bifurcation of the left middle cerebral artery in postoperative CTA, no stenosis of the parent artery or branch was observed, and an untreated aneurysm at the bifurcation of the right middle cerebral artery was found (red arrow). (**O**) Surgical incision and bone window.

A 60 years female patient admitted the hospital due to 10 days of intracranial aneurysm detected during cerebral infarction examination. She had a history of hypertension for 8 years and a history of 2 ischemic attacks. After admission, head and neck CTA showed bilateral bifurcation aneurysms in the middle cerebral artery. Based on the patient's history of two episodes of numbness on the right side of the face, as well as the presence of a large and irregular aneurysm on the left side, it is considered a high-risk aneurysm and has a priority level of treatment. After a thorough evaluation of the surgical plan and risks, the feasibility of the surgery was evaluated using 3D Slicer reconstruction virtual reality technology. Finally, the left supraorbital eyebrow keyhole approach was chosen for surgical treatment, and the aneurysm was successfully clamped during the operation with good results. One week after surgery, a follow-up CTA of the head and neck showed good occlusion of the aneurysm in the left bifurcation of the brain, without delayed bleeding or stenosis of the parent artery and branches. After one month of outpatient follow-up, the patient had clear consciousness and no positive signs of the nervous system (Fig. 2A–O).

#### Case 3

**Figure 3:**
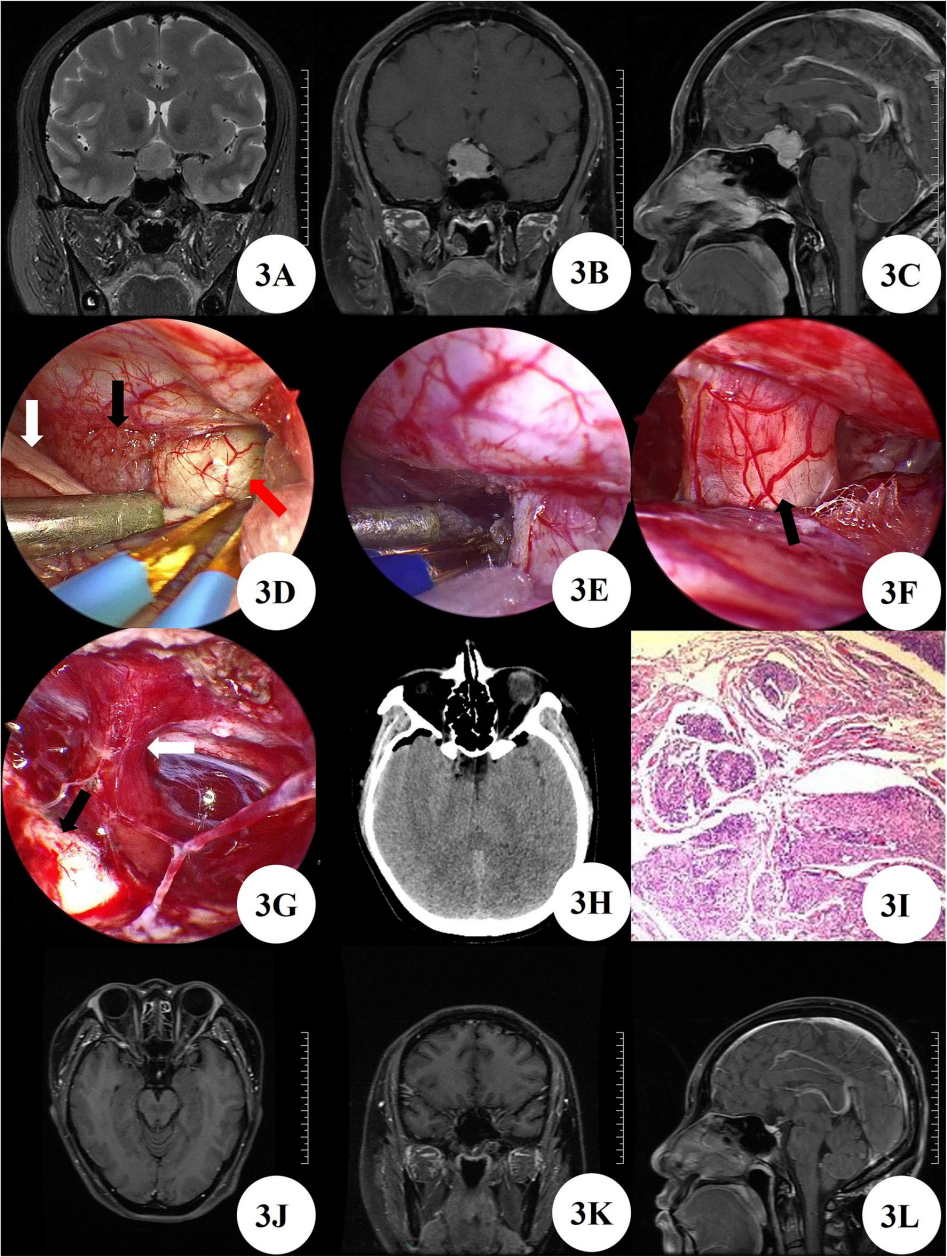
(**A**–**C**) Preoperative MRI shows mass lesions in the sellar region and suprasellar region. (**D**) Tumor (Black arrow), olfactory nerve (White arrow), and optic nerve visible (Red arrow) by neuroendoscopy. (**E**) Detachment of tumor base. (**F**) The right optic nerve is well preserved (Black arrow). (**G**) The pituitary stalk (White arrow) and left optic nerve (Black arrow) are well preserved. (**H**) Postoperative CT shows tumor resection with no bleeding in the surgical area. (**I**) Pathological examination diagnosed meningioma. (**J**–**L**) Follow up MRI at 8 months post-surgery showed no recurrence of the tumor.

A 36 years female patient admitted the hospital due to 2 years of headache with decreased vision and worsening for 1 month. After admission, MRI enhancement of the sellar region showed a mass in the sellar region, indicating a high possibility of meningioma. The right internal carotid artery cavernous sinus segment and bilateral anterior cerebral artery A1 segment were enveloped. The patient had a space occupying lesion in the anterior skull base and underwent minimally invasive surgery through the supraorbital eyebrow arch keyhole approach by neuroendoscopy, with good prognosis. Pathological examination diagnosed meningioma. Postoperative CT and enhanced MRI of the sellar region at 8 months confirmed complete resection without delayed bleeding, tumor residue or recurrence. After 8 months of outpatient follow-up, the patient had clear consciousness and no positive signs of the nervous system (Fig. 3A–L).

#### Case 4

**Figure 4:**
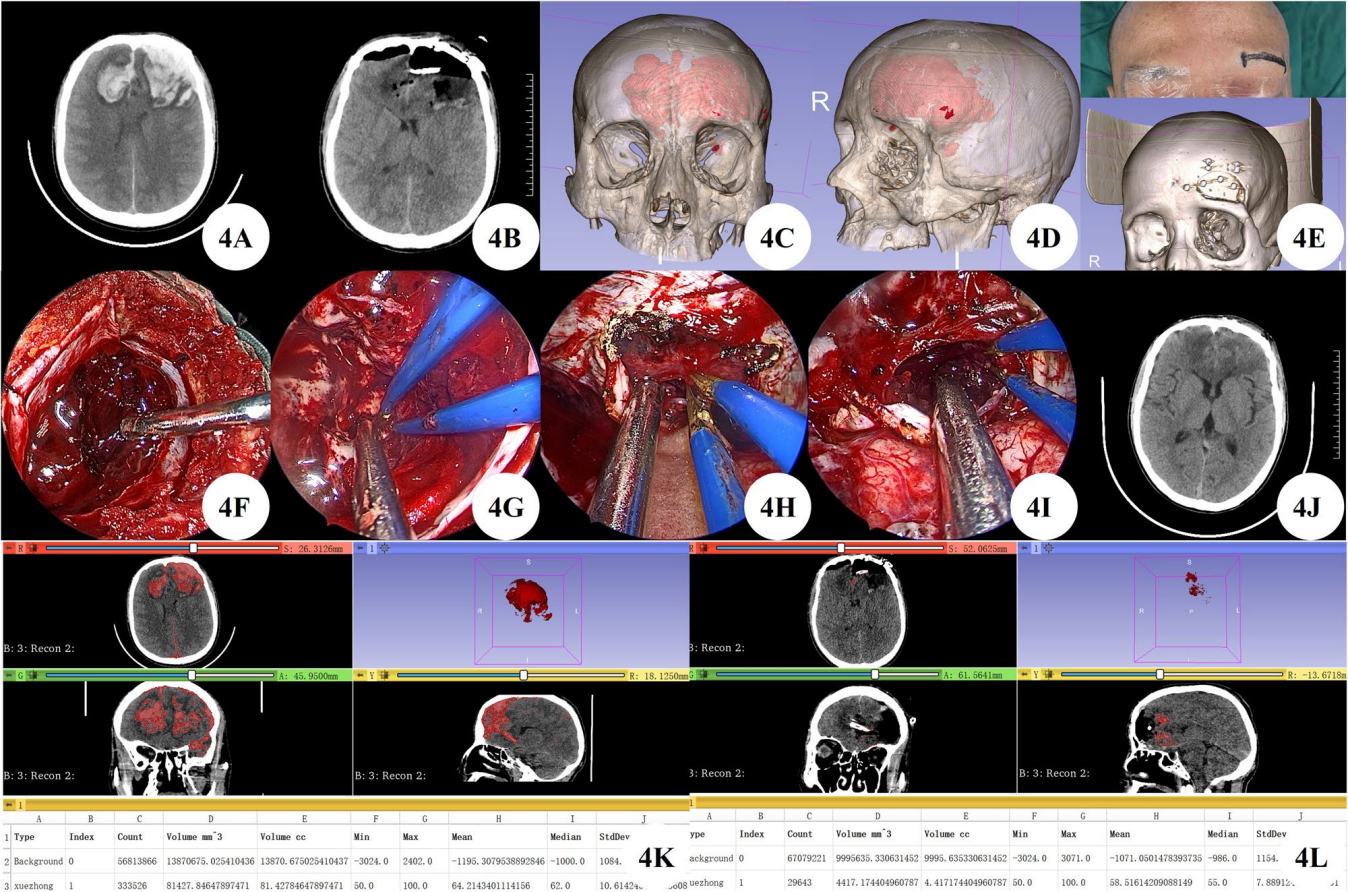
(**A**) Preoperative CT shows bilateral frontal lobe contusion and hematoma formation. (**B**) Postoperative CT shows bilateral frontal lobe brain contusions and hematoma cleared completely. (**C**,**D**) 3D Slicer reconstruction of virtual reality images of hematoma for preoperative evaluation. E: Surgical incision and bone window. (**F**,**G**) Clearing ipsilateral brain contusion and hematoma by neuroendoscopy. (**H**,**I**) Neuroendoscopic incision of cerebral falx and removal of contralateral brain contusion and hematoma. (**J**) 20 days post-surgery, CT scan showed good recovery. (**K**–**L**) 3D Slicer accurately calculates preoperative and postoperative hematoma volume.

A 46 years male patient admitted to the hospital due to being found to have a consciousness disorder for more than 10 h. The patient's family informed the patient that they had a head injury 2 days before admission, and there were no other special symptoms except for mild headache, so they did not seek medical treatment. Upon admission, emergency cranial CT showed hematoma formation in the left temporal lobe and bilateral frontal lobe, subarachnoid hemorrhage, left frontal epidural hematoma, and occipital bone fracture. The patient suffered from bilateral frontal lobe brain contusion and hematoma formation. The 3D Slicer accurately calculated the hematoma volume to be 81.43 ml, indicating a consciousness disorder and clear surgical indications. However, the patient did not have any brain herniation. A comprehensive evaluation of the surgical plan was conducted, and no skull removal decompression was required. The 3D Slicer reconstructed a virtual reality image of the hematoma, and the feasibility of the eyebrow arch keyhole surgery under simulation endoscopy was preliminarily evaluated. After communicating the surgical plan with the patient's family, they chose to undergo eyebrow arch keyhole surgery. By neuroendoscopy, the left supraorbital eyebrow arch keyhole approach was performed to remove bilateral frontal lobe brain contusions and intracerebral hematoma. Postoperative CT confirmed that the hematoma was completely cleared, and 3D Slicer was used to accurately calculate the hematoma volume of approximately 4.42 ml, with a clearance rate of 94.57%. After one month of outpatient follow-up, the patient had clear consciousness and no positive signs of the nervous system (Fig. 4A–L).

#### Case 5

**Figure 5:**
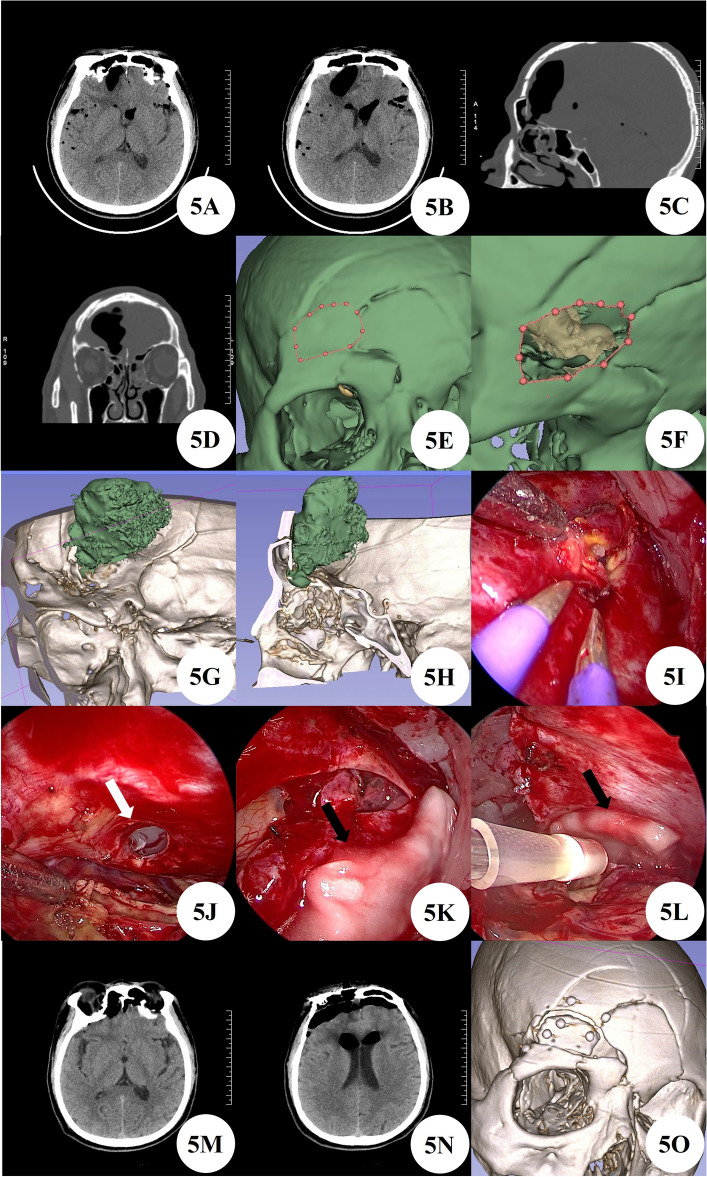
(**A**–**D**) Preoperative brain CT findings of intracranial gas accumulation and comminuted skull fractures. (**E**,**F**) Preoperative 3D Slicer reconstruction and simulation of surgical approach to evaluate the feasibility of this surgical approach. (**G**,**H**) Further evaluation of the location of bone fistula using preoperative 3D Slicer reconstruction. (**I**) Intraoperative neuroendoscopic findings of fistula and brain tissue swelling. (**J**) After thorough cleaning, obvious fistula can be seen (white arrow). (**K**,**L**) Multilayer artificial meninges and biological glue repair (black arrow). (**M**,**N**) Postoperative brain CT reexamination. (**O**) Postoperative 3D Slicer reconstruction of actual bone window.

A 56 years male patient admitted to the hospital due to cerebrospinal fluid rhinorrhea after head injury for over a month. Upon admission, the brain CT revealed multiple intracranial gas accumulation, with a gas containing cavity formed in the right frontal lobe, and multiple skull fractures in the craniofacial bone and anterior cranial fossa floor. We used 3D Slicer reconstruction for preoperative planning and still used the eyebrow keyhole approach for minimally invasive surgery. During the surgery, multiple fistulas were visible by neuroendoscopy, and bubbles emerged at the fistulas. The dura mater and artificial meninges of the fistulas were sutured and repaired with biological glue. Postoperative follow-up of brain CT and 3-month outpatient follow-up showed no recurrence of cerebrospinal fluid leakage or other complications (Fig. 5A–O).

### Ethical approval

All patients or family members have signed informed consent forms for study participation. All procedures performed in studies involving human participants were in accordance with the ethical standards of the institutional and/or national research committee and with the 1964 Helsinki declaration and its later amendments or comparable ethical standards. This study was approved by the ethics committee of Clinical Research, Our Hospital (WDRY2023-K139).

## Results

The good prognosis rate of intracranial aneurysms was 17/21 (81%), and the symptom improvement rate of intracranial space occupying lesions was 8/9 (88.9%). Among them, the initial symptoms of one patient with no improvement were not related to space occupying, while the total effective rate of the other three types of patients was 9/9 (100%). The length of the skin incision for all patients is about 4 cm. All patients were statistically analyzed for bone window size, length of hospital stay and postoperative length of hospital stay, preoperative neurological symptoms, and neurological symptoms obtained from outpatient or telephone follow-up one month after surgery. Further analysis of the location of intracranial aneurysms, preoperative Hunt Hess score, and modified Fisher score in patients with intracranial aneurysms; Further analysis of intracranial space occupying lesions, including tumor pathological type and grading, lesion size and location; Further analysis of the location of bleeding, presence of brain herniation, preoperative and postoperative hematoma volume (hematoma clearance rate) in patients with traumatic brain injury and cerebral hemorrhage. The average length of the craniotomy bone window of the supraorbital eyebrow arch keyhole is 3.77 ± 0.31 cm, and the average width is 2.53 ± 0.23 cm. The average hospital stay for all patients was 18.77 ± 7.00 days, and the average postoperative hospital stay was 14.77 ± 6.59 days. The average clearance rate of hematoma by neuroendoscopy is 95.00% ± 1.51%. The detailed information of all patients mentioned above is shown in Tables 1, 2, and 3.

**Table 1:** Clinical data of aneurysms.

Number	Gender	Age	Location of aneurysm	Aneurysm size (cm * cm)	Hunt-Hess grading	modified Fisher grade	External ventricular drainage (Y/N)	Bone window area (cm * cm)	hospitalization time (day)	Postoperative hospitalization time (day)	Preoperative neurological symptoms	Neurological symptoms 1 month after surgery	Notes
1	F	60	Anterior communicating artery aneurysm	2.3 * 2.4	0	0	N	3.0 * 3.6	23	15	Right head and face numbness, headache	Normal	
2	M	62	Anterior communicating artery aneurysm	6 * 2	0	0	N	2.8 * 3.8	28	14	Normal	Normal	
3	F	76	Anterior communicating artery aneurysm	8.8 * 7.0	3	1	N	2.3 * 3.4	15	11	Headache, vomiting, drowsiness, GCS = 14	Normal	
4	F	67	Right A1 and A2 aneurysms (multiple)	2.4 * 2.3	0	0	N	2.5 * 3.5	15	10	Headache	Normal	
				3.8 * 4.6									
5	M	60	Anterior communicating artery aneurysm	2.7 * 4.1	0	0	N	2.8 * 3.8	14	8	Normal	Normal	
6	M	60	Anterior communicating artery aneurysm	7.8 * 9.5	0	0	N	3.2 * 4.2	14	11	Right limb weakness	Normal	
7	M	55	Anterior communicating artery aneurysm	7.4 * 3.8	4	2	Y	2.6 * 3.3	17	17	Severe headache, vomiting, light coma, GCS = 8	Deep consciousness coma, death	Concurrent hydrocephalus, emergency ventricular drainage surgery, postoperative death due to sudden cardiac arrest
8	F	60	Right A1 aneurysm	2.2 * 3.1	0	0	N	2.3 * 3.5	12	9	Intermittent headache	Normal	
9	F	53	Left A2 aneurysm	3.4 * 3.6	3	2	Y	2.8 * 3.3	12	12	Severe headache, sweating, drowsiness, GCS = 14	Normal	
10	F	59	Left posterior communicating artery aneurysm	3.1 * 3.4	0	0	N	2.3 * 3.8	16	10	Intermittent dizziness	Normal	
11	F	52	Left posterior communicating artery aneurysm	2.9 * 3.0	0	0	N	2.6 * 4.2	16	11	Intermittent headache	Normal	
12	F	61	Anterior communicating artery aneurysm	3.7 * 4.7	3	2	Y	2.3 * 3.7	12	12	Headache, vomiting, drowsiness, GCS = 14	Normal	
13	F	51	Right A2 aneurysm (multiple)	3.8 * 4.0	4	4	Y	2.4 * 3.9	14	13	Moderate coma, limb tingling and retraction, GCS = 5	Mental confusion, GCS = 13	
				2.3 * 1.1									
14	F	47	Left A1 aneurysm (left basal ganglia hemorrhage)	4.6 * 5.8	0	0	N	2.7 * 4.2	14	7	Right limb weakness, unstable standing	Normal	
15	F	55	Anterior communicating artery aneurysm	1.8 * 2.7	1	4	N	2.6 * 3.8	31	30	Headache	Mental confusion, GCS = 12	Two days after surgery, there was right pupil dilation and hydrocephalus, and recovery was achieved after performing ventricular drainage surgery
16	M	34	Anterior communicating artery aneurysm	2.5 * 3.2	1	2	Y	2.4 * 4.0	29	29	Rupture of aneurysm after head injury, severe headache	Normal	
17	F	65	Left A2 aneurysm	2.0 * 4.2	2	2	Y	2.5 * 3.8	22	21	Severe headache, nausea and vomiting, GCS = 13	Normal	
18	M	49	Anterior communicating artery aneurysm; Right P1 aneurysm	2.3 * 3.5	2	1	Y	2.5 * 3.8	33	32	Severe headache, nausea and vomiting	Normal	One hospitalization and two surgeries, craniotomy and clipping surgery for anterior communicating artery aneurysm; Interventional embolization surgery for right P1 aneurysm
				1.7 * 1.6									
19	M	42	Anterior communicating artery aneurysm	2.9 * 2.5	2	3	N	2.4 * 3.6	22	21	Severe Headache	Normal	
20	F	60	Bilateral M1 aneurysms in the brain	8.4 * 5.6 (L)	0	0	N	2.6 * 3.6	15	11	Minor cerebral infarction examination unexpectedly discovers intracranial aneurysm	Normal	
				6.0 * 4.6 (R)									
21	F	66	Anterior communicating artery aneurysm	2.8 * 1.7	0	0	N	2.1 * 3.0	39	13	Light consciousness coma, GCS = 8 T	Light consciousness coma, GCS = 8 T	One hospitalization and two surgeries, ventricular hemorrhage and hematoma removal surgery, followed by another operation of aneurysm clipping surgery after the condition stabilized

**Table 2:** Clinical data of space occupying lesions.

Number	Gender	Age	Main diagnosis	WHO Level	Lesion size (cm * cm * cm)	Location of lesion	Bone window area (cm * cm)	hospitalization time (day)	Postoperative hospitalization time (day)	Preoperative neurological symptoms	Neurological symptoms 1 month after surgery
1	F	47	Olfactory sulcus meningioma	1	3.0 * 3.3 * 2.5	Middle of anterior skull base	2.5 * 4.0	10	6	Intermittent head pain with anosmia	anosmia
2	F	36	suprasellar meningioma	1	2.2 * 1.7 * 1.9	Suprasellar	2.7 * 4.2	16	13	Headache with decreased vision	Visual improvement
3	F	59	Anterior skull base meningioma	1	1.9 * 2.0 * 2.0	Beside the midline of the left frontal lobe	2.6 * 3.8	11	10	Headache and dizziness	Normal
4	F	57	Nodular meningioma of saddle	1	1.5 * 2.0 * 2.0	Middle of anterior skull base	2.8 * 3.6	14	12	Dizziness and blurred vision	Normal
5	F	66	Supraorbital meningioma	1	1.7 * 1.7 * 1.3	Right supraorbital posterior part	2.4 * 3.7	11	8	Binaural hearing loss	Binaural hearing loss
6	F	58	Olfactory sulcus meningioma	1	2.2 * 3.0 * 3.5	Olfactory groove	2.7 * 4.0	24	9	Insomnia with periorbital pain	Symptom improvement
7	F	65	Suprasellar space occupying lesion	Rathke cyst	1.0 * 0.7 * 1.0	Suprasellar	2.6 * 4.0	21	14	Dizziness with decreased vision	Visual improvement
8	M	29	Orbital space occupying lesions	Dermoid cyst	2.3 * 1.8 * 3	Left frontal lobe	2.3 * 4.0	13	10	Post traumatic headache with discovery of intracranial mass	Normal
9	M	44	Anterior skull base meningioma	Atypical meningioma; WHO Level 2	2.8 * 2.1 * 2.7	Left frontal region	2.5 * 3.5	24	14	Headache, nausea	Normal

**Table 3:** Clinical data of the other patients.

Number	Gender	Age	Main diagnosis	Cerebral hernia (Y/N)	Preoperative hematoma volume (ml)	Postoperative hematoma volume (ml)	Hematoma clearance rate	Bone window area (cm * cm)	hospitalization time (day)	Postoperative hospitalization time (day)	Preoperative neurological symptoms	Neurological symptoms 1 month after surgery
1	M	58	Right frontal lobe hemorrhage breaks into the ventricle	Y	80.2	2.4	97.01%	2.4 * 4.2	13	13	Light consciousness coma, bilateral pupils 2.5 mm, GCS = 7, left muscle strength level 0, right muscle strength level 4	Normal
2	M	50	Cerebrospinal fluid rhinorrhea (spontaneous)	–	–	–	–	2.6 * 4.0	20	17	Cerebrospinal fluid rhinorrhea	Normal
3	F *	58	Left frontal lobe brain contusion and laceration with hematoma	Y	52.8	3.3	93.75%	2.5 * 3.4	32	32	Light consciousness coma, bilateral pupils 2.5 mm, GCS = 6 T	Normal
4	M	57	Bilateral frontal lobe brain contusion and laceration with hematoma	Y	62.5	2	96.80%	2.9 * 4.2	16	16	Headache	Normal
5	F	68	Right frontal lobe brain contusion and laceration with hematoma	Y	72	4.5	93.75%	2.5 * 3.8	20	20	Drowsy, bilateral pupils 2.5 mm, GCS = 10, limb muscle strength level 3	Normal
6	F	76	Right frontal lobe brain contusion and laceration with hematoma	N	23.8	1.4	94.12%	2.2 * 3.3	16	16	Light consciousness coma, bilateral pupils 2.5 mm, GCS = 8	Normal
7	M	56	Cerebrospinal fluid rhinorrhea	–	–	–	–	2.1 * 3.5	16	13	Cerebrospinal fluid rhinorrhea	Normal
8	F	63	Cerebrospinal fluid rhinorrhea	–	–	–	–	2.4 * 4.2	24	18	Cerebrospinal fluid rhinorrhea	Normal
9	M	46	Bilateral frontal lobe brain contusion and laceration with hematoma	Y	81.43	4.42	94.57%	2.4 * 3.8	18	18	Light consciousness coma, bilateral pupils 2.5 mm, GCS = 9	Normal

*Explanatory note After performing left temporal parietal lobe hematoma removal surgery and temporal parietal skull decompressive craniectomy in an external hospital, delayed left frontal lobe brain contusion and hematoma formation occurred. The patient was transferred to our hospital for emergency surgical treatment. After the intracranial condition recovered, the patient underwent right tibiofibular fracture open reduction and external fixation surgery again.

## Discussion

With the development of micro neurosurgery and neuroendoscopy technology, people's understanding of side injuries in craniotomy surgery is also increasing. Large surgical incisions, damage to normal brain tissue structures, brain tissue atrophy, damage to neurovascular structures, and longer postoperative recovery time are considered urgent changes that need to be made. Over the past few decades, neurosurgeons have been dedicated to the research of “minimally invasive” surgery. The term “minimally invasive” surgery is a conceptualization of surgical techniques, which not only requires minimizing additional damage to the target area, but also requires attention to avoid unnecessary damage to the intracranial and extracranial tissues during the surgical approach process. Hippocrates emphasized the importance of minimizing iatrogenic trauma during intervention over 2000 years ago. From a philosophical perspective, the less tissue is destroyed, the less tissue needs to be healed; The less exposed the brain, the less damaged the brain; The smaller the surgical pathway, the fewer tissues and functional structures at risk^1^. Based on the above requirements, with the unremitting efforts of neurosurgeons, the concept of “keyhole” has been proposed, which utilizes modern surgical technology to develop the minimum traumatic approach that allows for normal surgical operations, while achieving maximum surgical results while ensuring safety. The concept of “keyhole approach” surgery has greatly promoted the development of this field, improved surgical efficiency and safety, reduced the incidence of various postoperative complications, and accelerated the rehabilitation process. At present, the main keyhole surgical approaches include supraorbital eyebrow arch keyhole craniotomy, supraorbital lateral craniotomy, intraorbital keyhole approach, lesser pterygoid keyhole craniotomy, mini orbitozygomatic craniotomy, and lesser anterior longitudinal fissure approach^1,6^. However, these minimally invasive surgeries have inherent defects such as narrow field of view and low deep illumination, and the emergence of neuroendoscopy can precisely compensate for the shortcomings in such surgeries. Neuroendoscopy has the advantages of good illumination, flexible degrees of freedom, and close observation^7^. It can provide high-intensity illumination and a broad surgical field for the deep surgical area of minimally invasive surgery. It can reach the target area through narrow surgical channels and display deeper pathological tissues with high resolution. Research^8^ has shown that a combination of neuroendoscopy, image-guided systems, and intraoperative assistive devices (such as ultrasound and magnetic resonance imaging) can aid in precise surgical localization and complete lesion resection through a keyhole approach. The supraorbital eyebrow arch keyhole approach under transcranial neuroendoscopy has gradually become one of the popular surgical treatments for anterior and middle skull base diseases. The advantage of this method is that it is minimally invasive and utilizes gravity to naturally sag the frontal lobe brain tissue, directly reaching the deep target area of the skull base through the subdural space and avoiding secondary damage caused by brain tissue fistula. With the flexible degrees of freedom of transcranial neuroendoscopy and the assistance of multiple angle working lenses, a deep surgical area several times wider than the surgical entrance can be obtained.

The supraorbital eyebrow arch keyhole approach, due to its minimally invasive surgical opening and cone-shaped enlarged surgical area, directly reaches the skull base and brain pools such as the optic chiasm pool, carotid artery pool, and vertebral plate through the natural space under the dura mater of the anterior skull base to release cerebrospinal fluid drainage, thereby obtaining sufficient surgical space. It is widely popular in craniotomy surgeries for anterior circulation aneurysms, anterior skull base tumors, and suprasellar tumors. A cadaveric autopsy study^9^ showed that under 0° endoscopy, the olfactory bulb, olfactory tract, bilateral optic nerve, optic chiasm, bilateral anterior cerebral artery segments A1 and A2, anterior communicating artery, internal carotid artery, middle cerebral artery, origin of posterior communicating artery, and anterior choroid artery can be exposed. Under 30° endoscopy, the funnel, sellar septum, and upper and lower trunk branches of the M2 segment of the middle cerebral artery can be exposed. Endoscopy is inserted into the interspinous cistern between the bifurcation of the internal carotid artery and the oculomotor nerve, which is sufficient to expose structures such as the basal artery and some posterior cerebral arteries. However, due to severe atrophy of brain tissue after formalin fixation in autopsy, there may be significant differences in the actual exposure range compared to live anatomy, especially in cases of brain tissue swelling or large intracranial masses after arterial aneurysm hemorrhage, the exposure of the surgical area will be more difficult. The advantage of the neuroendoscopic approach lies in the instrument itself, as it is movable and can be equipped with lenses from different angles, providing a panoramic view for the supraorbital microsurgical approach. In order to utilize these attributes and be able to perform dual handed microsurgical operations simultaneously, it may be necessary for another surgeon to assist in controlling the neuroendoscopy, who needs to ensure the best field of view for the surgeon while avoiding collisions with other instruments^10^. But not all surgeries are suitable for the supraorbital eyebrow arch keyhole approach by neuroendoscopy. Only by selecting the appropriate case for the above surgery under the premise of clear surgical field and sufficient surgical space can the safety of patients be guaranteed. Therefore, some scholars advocate the use of neuroendoscopy for microsurgical neurosurgery via the supraorbital eyebrow arch keyhole approach only in unruptured intracranial aneurysms or aneurysms that rupture after absorption of subarachnoid hemorrhage^1^.

Our team completed 21 cases of anterior communicating artery aneurysm, anterior cerebral artery aneurysm, middle cerebral artery aneurysm, and posterior communicating artery aneurysm through the eyebrow arch keyhole approach by neuroendoscopy. All aneurysm surgeries require dissection of the optic chiasmal pool, carotid artery pool, and opening of the endplate to release cerebrospinal fluid for decompression, in order to obtain sufficient surgical space. For cases of ruptured and bleeding aneurysms with severe brain tissue swelling that cannot enter the anterior skull base through the subdural space, we puncture the lateral ventricle in the surgical area and place a tube to release cerebrospinal fluid for decompression before entering the anterior skull base, which can fully expose the surgical field of view. Our clinical research results on the feasibility of using this surgical approach to treat intracranial aneurysms are similar to those of Van Lindert et al.^11^. Meanwhile, they also pointed out that clipping the contralateral internal carotid artery aneurysm is safe unless the aneurysm is located on the dorsal side or obstructed by the anterior clinoid process. For the P1 segment aneurysm of the posterior cerebral artery and the tip aneurysm of the basilar artery, they also believe that it can be theoretically reached and clipped, but considering the narrow surgical pathway and the uncontrollability of deep operations, they do not recommend this surgical procedure.

Anterior skull base tumors are mainly caused by their occupying effect, which compresses important structures such as the adjacent olfactory nerve, optic nerve, vascular structure, and frontal lobe gyrus, leading to positive neurological symptoms. Although traditional coronal incisions through the anterior skull base or pterional approach have achieved good results in removing these tumors, due to the large surgical trauma, long surgical time, and long postoperative recovery time Poor cosmetic effects and neurological complications have forced neurosurgeons to explore more minimally invasive surgical methods^12^. A large number of studies^8,13–17^ have shown that the supraorbital eyebrow arch keyhole approach by neuroendoscopy is a good choice. Our team used the above method to complete 9 cases of anterior skull base tumors, including supraorbital meningioma, olfactory sulcus meningioma, supraorbital epidermoid cyst, and suprasellar mass. Except for a transient diabetes insipidus in the suprasellar mass, all patients had no significant neurological complications. Our research results show that with the assistance of different angles of neuroendoscopy, the skull base lesions and surrounding nerve and vascular structures can be clearly displayed, especially the occluded deep lesions that cannot be observed under the direct light field of the microscope. Its advantages of close observation and flexible degrees of freedom provide high-definition, close range, and almost all-round observation capabilities. Therefore, we believe that the neuroendoscopic supraorbital eyebrow keyhole approach is a promising approach for anterior skull base surgery.

The endoscopic approach through the eyebrow arch keyhole is increasingly being used in the surgical treatment of skull base diseases^16^. In addition to its application in the surgery of intracranial aneurysms and brain tumors, our team has also attempted this surgical approach to treat anterior skull base diseases such as frontal lobe brain contusion and laceration, cerebrospinal fluid rhinorrhea, and has achieved good results. The advantages of good lighting, flexible degrees of freedom, and close observation of neuroendoscopy have been well demonstrated in the process of hematoma removal. With the use of working lenses at 0° and 30° angles, even the unilateral eyebrow arch keyhole approach can reach the bilateral anterior skull base to remove hematoma. Our research shows that the supraorbital eyebrow arch keyhole approach has significant advantages in surgical time and blood loss for the removal of frontal and basal brain contusions and hematomas. For elderly patients with frontal and basal brain contusions, this may be a good choice. However, further prospective multicenter clinical research and evaluation are needed to determine the indications and timing of surgery. The current popular surgical method for anterior skull base cerebrospinal fluid rhinorrhea is endoscopic repair of cerebrospinal fluid leakage through the nasal cavity^18^. We choose the neuroendoscopic approach through the eyebrow arch keyhole, which uses gravity to make the frontal lobe brain tissue naturally sag and reach the damaged fistula site through the natural subdural space for repair surgery. Compared with transnasal surgery, it solves the problem of requiring multi-instrument surgical operations in narrow surgical channels; At the same time, the upstream repair method for intracranial repair is significantly better than the leak blocking downstream repair method for extracranial repair.

Of course, the supraorbital eyebrow keyhole approach by neuroendoscopy also has its limitations. Neuroendoscopy is most suitable for use with reasonable control of bleeding and does not require highly technically challenging manual operation, as it takes up some space, especially with the tilted field of view provided by endoscopes at a certain angle, making the operation even more difficult; Therefore, endoscopy can become a powerful tool for entering blind spots, but improper use may worsen the damage. Akçakaya et al.^9^ showed that the length of skin incision through the supraorbital eyebrow arch keyhole approach was 3.4–4 cm, with an average of 3.68 ± 0.19 cm. The average length of the bone window is about 2.65 ± 0.23 cm (2.2-3 cm), and the average width is about 1.43 ± 0.12 cm (1.3–1.7 cm), which also limits the surgical operation of two people, multiple hands, and multiple instruments to a certain extent. Therefore, our bone window has an average length of 3.77 ± 0.31 cm and an average width of 2.53 ± 0.23 cm, slightly larger than the bone window area of Akçakaya et al. In addition, due to the lateral area of the endoscope not being within the surgical field of view, there may be a "black under the lamp" situation, and adjusting the angle of the endoscope may accidentally damage any key structures within the surgical channel, such as the optic nerve, perforating vessels, bridging veins, brain tissue, etc^1^. Meanwhile, due to the different development of the frontal sinus in each patient, the incidence of accidental frontal sinus opening is about 18%^15^. Improper management of frontal sinus opening can increase the risk of postoperative infection.

## Summary

Neuroendoscopic minimally invasive surgery through the supraorbital eyebrow arch keyhole approach is a relatively new technique. It not only has minimal trauma and is aesthetically pleasing, but can also enter the intracranial area including the anterior cranial fossa, middle fossa, and the lateral side of the cavernous sinus. It basically covers all anterior and partial middle cranial base surgeries. Our results indicate that endoscopic surgery through the supraorbital eyebrow arch keyhole approach is safe and effective for the treatment of anterior skull base lesions and cerebral hemorrhage. However, this retrospective study is a single center, small sample study, and the good surgical results do not exclude the subjective screening of suitable patients by clinical surgeons, which may have some bias. Although the clinical characteristics such as indications and contraindications of this surgical method still require further prospective and multi neutral clinical research validation, our study still provides a new approach and choice for minimally invasive surgical treatment of anterior skull base lesions.

## Data Availability

The datasets used and/or analysed during the current study available from the corresponding author on reasonable request.
